# From 2D to 3D: novel nanostructured scaffolds to investigate signalling in reconstructed neuronal networks

**DOI:** 10.1038/srep09562

**Published:** 2015-04-24

**Authors:** Susanna Bosi, Rossana Rauti, Jummi Laishram, Antonio Turco, Davide Lonardoni, Thierry Nieus, Maurizio Prato, Denis Scaini, Laura Ballerini

**Affiliations:** 1 Department of Chemical and Pharmaceutical Sciences, University of Trieste, Trieste-Italy; 2 Life Science Department, University of Trieste, Trieste-Italy; 3 Department of Neuroscience and Brain Technologies Italian Institute of Technology (IIT), Genoa-Italy; 4 ELETTRA Synchrotron Light Source, Trieste-Italy; 5 International School for Advanced Studies (SISSA), Trieste-Italy

## Abstract

To recreate *in vitro* 3D neuronal circuits will ultimately increase the relevance of results from cultured to whole-brain networks and will promote enabling technologies for neuro-engineering applications. Here we fabricate novel elastomeric scaffolds able to instruct 3D growth of living primary neurons. Such systems allow investigating the emerging activity, in terms of calcium signals, of small clusters of neurons as a function of the interplay between the 2D or 3D architectures and network dynamics. We report the ability of 3D geometry to improve functional organization and synchronization in small neuronal assemblies. We propose a mathematical modelling of network dynamics that supports such a result. Entrapping carbon nanotubes in the scaffolds remarkably boosted synaptic activity, thus allowing for the first time to exploit nanomaterial/cell interfacing in 3D growth support. Our 3D system represents a simple and reliable construct, able to improve the complexity of current tissue culture models.

Modern neuroscience increasingly relies on material science tools to engineer 3D neuronal network models *in vitro*^1^,^2^,^3^,^4^. Most studies in this area are dedicated to the fabrication of biocompatible supports able to guide stem cell differentiation and growth into organized neuronal circuits^5^,^6^. In a parallel approach, growth supports are also engineered to obtain *in vitro* neural circuits, build up by primary brain cells, displaying a genuine 3D architecture. Allowing neurons to reconstruct synaptic networks in appropriate space coordinates and in the presence of homeostatic abilities expressed by neuroglia in the third-dimension may provide crucial insights into central nervous system (CNS) multilevel integration of signals in health and disease^1^,^2^,^3^,^4^. This promoted the emergence of a new generation of culture models aimed at mimicking tissue complexity *in vitro*, in particular 3D neuronal arborisation. For example, 3D cultures were developed by growing dissociated primary neurons on combined tiers of silica beads, used as cell growth surfaces^3^. In these systems the bottom layer of neurons was further interfaced to a 2D microelectrode array (MEA) to infer the impact of the multiple layers of interconnected neurons on the firing activity of the recorded cells^7^. Alternatively, sophisticated nanostructures were developed to allow neuronal growth in a true 3D, suspended fashion^8^. However, these systems lack in tissue-like features, such as porosity or elastic properties, preventing their translation into *in vivo* settings. Additionally, when analysing structure/function relations in neural circuits we need to isolate the impact of arranged layers of cells from that of 3D topology *per se*. Hydrogel based materials or 3D electrospun polymers have been used to obtain more realistic tissue constructs^9^,^10^. Yet, many of these systems are prone to degradation, in particular when in the presence of astrocytes^9^, while robustness in time is necessary to predict *in vitro* mechanisms of CNS development or disease. Ideally, when designing neuronal scaffolds, it is vital to manipulate the support features at the nanoscale. These are known to affect cell phenotype, or synapse construction, via resembling certain properties of the extra-cellular matrix (ECM)^11^,^12^. Here we report a biocompatible, synthetic polymer based-scaffold that can be reliably tailored in its mechanical micro- and nano-properties. Such scaffolds allowed the development of 3D hippocampal cultures where the soma and processes of neurons and neuroglia were exposed to the third dimension. The morphology of the 3D scaffold and that of the neuronal cultures were reconstructed by scanning electron (SEM) or confocal microscopy while patterns of activity were measured by simultaneously imaging the intracellular calcium activity of living neurons that differed only by their 2D or 3D geometries. Computational modelling of network dynamics, used to link network topology to network function, together with our experimental results, provide novel insights into how 3D morphology of connectivity affects neural circuit efficacy. We further nanostructured the scaffold by means of multi walled carbon nanotubes (MWCNTs), implementing the polymer with novel nano-topographies. This enabled for the first time to successfully transfer in a 3D construct the ability of MWCNTs to interface and boost cultured brain network activity^13^,^14^,^15^.

## Results

### Fabrication of scaffolds promoting 3D cell growth

**Figure 1:**
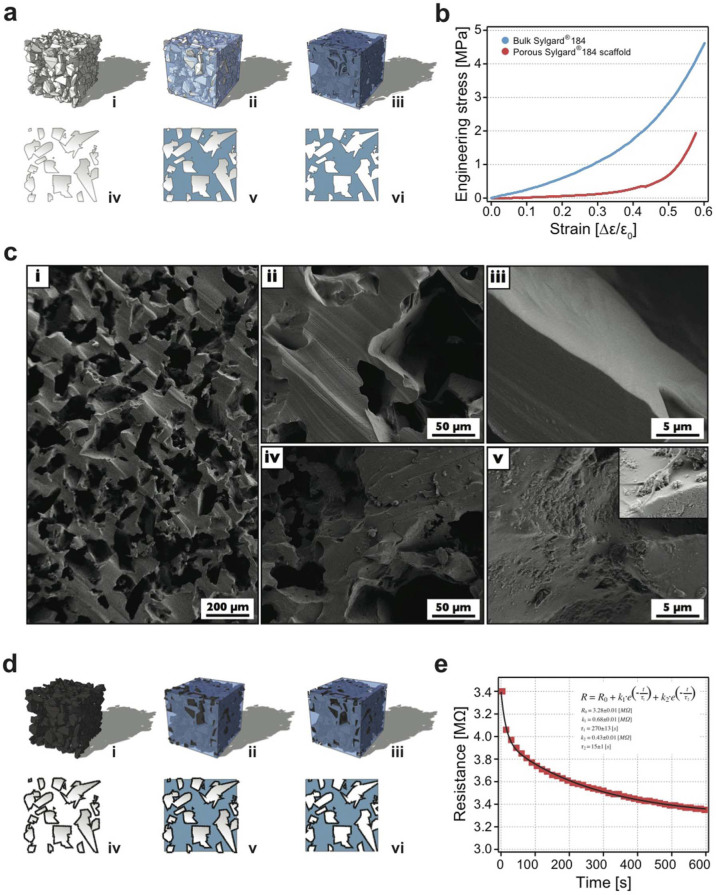
Scaffold fabrication and characterization. In (a) the fabrication steps of the PDMS scaffold are sketched: (i) the starting sugar mould (ii) uncured PDMS forced via a vacuum process within the mould (iii) after PDMS heat curing the sugar framework is dissolved leaving a self-standing PDMS replica; from (iv) to (vi) sketched cross sections of the previous steps. In (b) stress-strain plots for bulky PDMS (in blue) and porous PDMS scaffold (red). Elastic moduli were determined from the initial slope of the two corresponding stress-strain curves. In (c) SEM images of the PDMS based scaffolds: (i) low magnification SEM image of the PDMS scaffold. (ii) and (iv) higher resolution images of PDMS and PDMS-MWCNTs scaffolds, respectively; (iii) and (v) details of the previous images, note the exposed MWCNTs at the surface. Inset in (v) details a cell process in contact with the carbon nanotubes. In (d) sketched fabrication steps (as in (a)) of the MWCNTs modified PDMS scaffold. In (e) time dependent resistance measurement of a 5 mm side size cube of MWCNT endowed PDMS scaffold. Note the two exponential decay times of the measured resistance.

We develop a micro-porous, self–standing, elastomeric scaffold to implement 3D neuronal growth. The skeleton material of this structure is made by polydimethylsiloxane (PDMS) with micrometric cavities generated upon dissolving a sugar template that was previously embedded in PDMS (Fig. 1a). Scaffold fabrication procedure (see Methods) is an adaption and refinement of previous work^16^. PDMS elastomer has become a standard in microfabrication or microfluidics developments in general and, in particular, when dealing with biological applications, both in *in vitro* or *in vivo* contexts. This is due to the PDMS simple manufacturing together with its gas permeability, optical transparency, flexibility and the ease at which it can be shaped or modified even at the nanoscale during moulding^17^,^18^,^19^,^20^. In addition, the PDMS surface allows the chemical attachment or mechanical entrapment of nano/micro features in the moulding surface (e.g. nanoparticles^21^,^22^ nanowires^23^,^24^ or carbon nanotubes^16^,^25^). Besides, pristine PDMS surface is characterised by pronounced hydrophobicity, primary due to the presence of methyl chemical groups, this minus can be overtaken by oxygen plasma treatment, which turns the PDMS surface to hydrophilic by massively introducing hydroxyl groups^17^.

As sketched in Fig. 1a, PDMS scaffolds are the negative replica of a generating sugar framework, this allows to fabricate 3D structures with a spongy appearance characterized by pores of irregular shapes and dimensions, interconnected by random paths of connectivity. The diameter of the pores varies within the 20 ÷ 150 μm range (Fig. 1c i, ii) leading to 40% PDMS sponge final porosity with a corresponding bulk density of 0.58 g/cm^3^, as determined by gravimetric measurements. SEM (Fig. 1c) indicates that the sugar amount exceed percolation limit for such a system resulting in the generation of interconnected pores giving rise to intricate networks of channels within the PDMS scaffold.

Controlling the porosity of the material allows manufacturing its macroscopic stiffness, in that the wider the pores the lower the stiffness and *vice versa*^26^. For 3D hippocampal culture reconstruction, the scaffold's pores have to be large enough to allow permeation and migration of cells (neurons and neuroglia). On the other hand, this continuum of interconnected cavities should not be too large to phase out the scaffold's mechanical features. Our aim is to obtain a system truly allowing growth in 3D environment yet soft enough to match the viscoelastic nature of neural tissue. Young's modulus (E) values in rodent and human brains are estimated to be in the range of 0.1 to 20 kPa (1 nN/μm^2^ = 1 kPa)^27^. Using a compressive load-cell we measure, by uniaxial-load compression test (see Methods), an E value of 2.35 MPa for the bulk Sylgard® and of 45 kPa for the porous scaffold were obtained by calculating the initial linear slope of the stress-strain curve, (Fig. 1b).

We have recently shown the ability of MWCNTs to boost synaptic network activity and synapse formation when neurons are cultured interfaced to carbon nanotube growth supports^13^,^14^,^15^. To exploit interfacing MWCNTs in the three-dimensional neuronal circuits, we modify the PDMS porous scaffold. Within the same fabrication procedure described above, we generate micrometric cavities by a sugar mould previously mixed to MWCNTs (Fig. 1d; see Methods). This allows the formation of scaffolds with the pores layered by an irregular carbon nanotube carpet (around 100 nm thick; Fig. 1c iv and v) stably entrapped in the PDMS matrix. The porous environment is perfectly similar to that of control PDMS scaffolds (see SEM in Fig. 1c) except that MWCNTs are exposed on the holes' surfaces (Fig. 1c iii and v). The mechanical properties of the two scaffolds (control and MWCNTs) were measured (not shown) and the two materials do not display differences in compressibility. The electrical characterization (see Supplementary information) of the MWCNT enriched PDMS scaffold (Fig. 1e) reveals a low macroscopic conductivity of 10^−5^ S/cm, despite the presence of MWCNTs (whose electrical conductivity is in the order of 10^4^ S/cm^28^). This may result from the combined effects of the large contact resistances between contiguous carbon nanotubes on one hand and the porosity of the material on the other. These factors, together with the low volumetric fraction of MWCNTs, may prevent carbon nanotubes to reach their electrical percolation limit at the micro-scale. Interestingly, sample resistance measured at constant load (see Supplementary information) exhibits a time dependent behaviour characterized by a fast time decay constant (τ_f_) of 0.68 s and a slow time decay constant (τ_s_) of 15 s (Fig. 1e). Viscoelastic and porous-elastic properties of our material may induce time dependent stress relaxation forces that lead to a larger portion of the sample surface to contact the electrodes, thus resulting in a small decrease in the measured resistance (Fig. 1e).

We test the biocompatibility of both materials by culturing hippocampal cells in the two types of scaffolds (with and without MWCNTs). Such structures allow proper neuronal growth and development as documented by immunofluorescence staining and confocal microscopy (see below).

### Implementing 3D networks: confocal reconstruction of 2D and 3D hippocampal cultures

**Figure 2:**
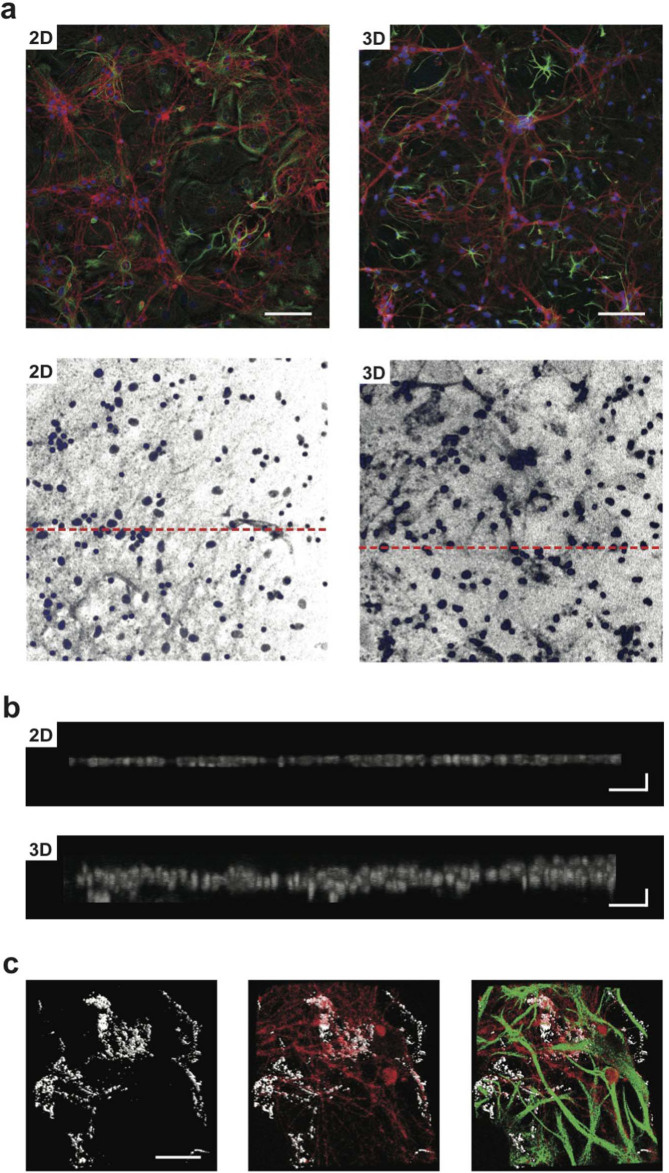
Development of primary neurons in 2D- or 3D-PDMS scaffolds. In (a) (top row) confocal micrographs show hippocampal cultures grown (9 DIV) on 2D-PDMS (left) and 3D-PDMS (right) immune-stained for β-tubulin III (in red), GFAP (green) and DAPI (blue). Scale bar: 100 μm. In the bottom rows images, only the DAPI channel is selected to highlight the nuclei under the two culturing conditions (same visual fields as in a. top); the dashed red lines represent the regions for which the z profile reconstructions are performed in (b) note the increased thickness of DAPI signal in the 3D-PDMS. Scale bar: 100 × 10 μm. In (c) confocal reconstruction of a 3D-MWCNT scaffold (left; in grey carbon nantubes are visualised by confocal under reflection mode acquisition, allowing to visualize the scaffold structure); confocal reconstruction of neurons (in red; middle) grown suspended within a pore and glia cells (in green; merged in right panel) acting as a support. Note the complex growth of neuronal and glial processes exposed to the third dimension. Scale bar: 50 μm.

In this set of experiments we use immunofluorescence techniques and confocal microscopy to compare neurons grown on 2D control substrates (named 2D-PDMS) with those grown on 3D scaffolds (named 3D-PDMS). To prove the formation *in vitro* of 3D cultures, we image by immunofluorescence the specific cytoskeletal components β-tubulin III, to visualize neurons, and glial fibrillary acidic protein (GFAP) to visualize astrocytes^15^,^29^,^30^,^31^,^32^. In all cultures tested (n = 24 for 2D and 3D), prior to confocal analysis, neuronal calcium activity was monitored (see below) and at the end of each recording session the samples were fixed for microscopy. Fig. 2a (top panels) shows low magnification confocal reconstructions where cultures display β-tubulin III positive cells (in red) and GFAP positive ones (in green) developed in the traditional planar (left) or in the novel 3D (right) supports, in both images nuclei are visualized by DAPI (in blue). In Fig. 2a (lower panels; same samples as in the top ones) only nuclei are visualised to disclose cell distribution as shown in Fig. 2b, where the rendering of 3D images from confocal Z stacks files is performed by zeta profile reconstruction. In Fig. 2b, the flat morphology of 2D-PDMS cultures is depicted, with cell nuclei distributed within a section almost corresponding to a monolayer of 13 μm thickness (on average 10 ± 5 μm, n = 10 cultures). Conversely, when similarly reconstructing the zeta profile in a 3D-PDMS section (dissected out from the 300 ÷ 400 μm total of the cultured 3D-PDMS sponge) the depicted thickness is of 60.4 μm (on average 60 ± 20 μm, n = 14 cultures). These observations indicate that neurons and glial cells in 3D-PDMS are disposed on different levels, as disclosed by their nuclei distribution (see also Supplementary movie-1). In Fig. 2c additional details of 3D cultures are visualised by zeta-stack reconstruction and image analysis. These 3D reconstructions reveal that neurons and neuronal processes are not constrained by the pre-build scaffold infrastructure and processes are also directly bridging one pore to the other. It is also possible to visualise β-tubulin III positive cell bodies suspended on glial cells with only their neuronal processes providing the necessary anchorage to the scaffold (Fig. 2c: Supplementary movie-2). These observations make our 3D model unique, in that cells are truly exposed to the third dimension while developing networks of connections, since the pore size of the scaffold facilitates the migration of cell bodies and/or the processes outgrowth virtually in all space coordinates.

### Imaging of calcium activity

**Figure 3:**
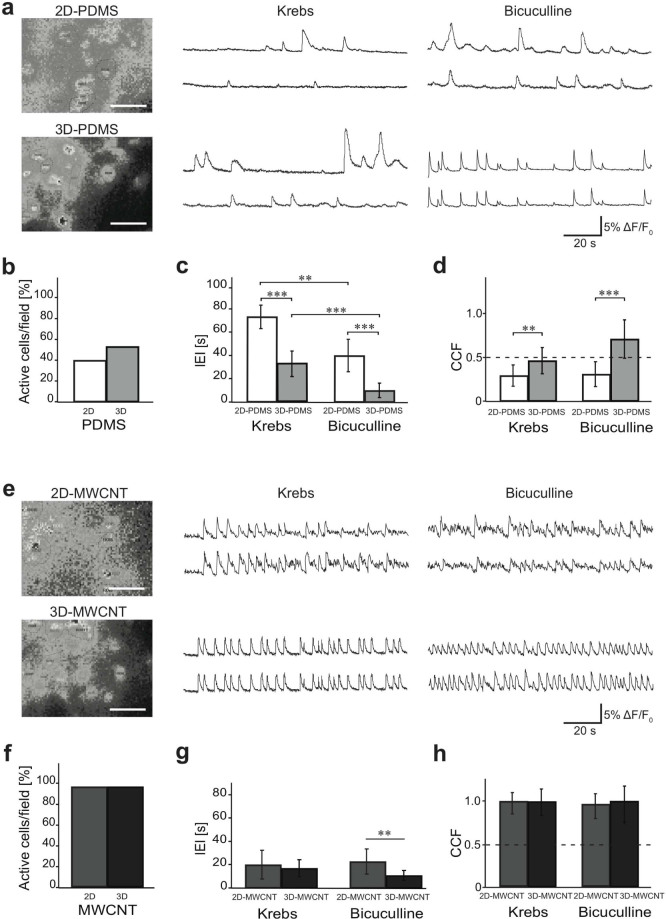
Network activity in 2D and 3D constructs. In (a) (left) snapshots of representative fields of neuronal cultures grown on 2D-PDMS (top) and 3D-PDMS (bottom) substrates, stained with the Oregon Green 488-BAPTA-1 AM. Dashed lines indicate the selected regions of interest (ROI). Scale bar: 50 μm. In (a) repetitive Ca^2+^-events spontaneously (middle) or bicuculline induced (right) recorded in hippocampal cultures of 9 DIV (two sample neurons were selected from the same field). In (b) histograms of the percentage of spontaneous active cells in 2D and 3D contexts. In (c) average of inter-event interval (IEI) values of 2D-PDMS and 3D-PDMS control cells (krebs) and disinhibited ones (bicuculline; ** = p < 0.01, *** = p < 0.001; two-way ANOVA tested with Holm–Sidak, data are mean ± SD). In (d) the histograms summarize the cross-correlation factors (CCF) measured under all experimental conditions (*Student's-t* test). In (e) (left) two snapshots representing recording fields in 2D-MWCNT and 3D-MWCNT. Scale bar: 50 μm. In (e) repetitive Ca^2+^ activities spontaneously (middle) or bicuculline induced (right) recorded in 2D- and 3D-MWCNT. Notably, 98% percentage of cells is spontaneously active in the presence of MWCNT, as shown in the plot in (f) In (g–h) the histograms summarize the IEI and CCF measured under all experimental conditions (two-way ANOVA tested with Holm–Sidak and *Student's-t* test when appropriate, data are mean ± SD).

To investigate the interplay between the 3D neuronal architecture and network dynamics, we monitor neuronal activity with fluorescence calcium imaging. Neurons, when cultured in 2D-PDMS or in 3D-PDMS, develop their connectivity in large areas (Fig. 2a). With our imaging set up we sampled representative regions of 120 × 160 μm^2^. Neurons stained with the membrane permeable Ca^2+^ dye Oregon Green 488 BAPTA-1 are simultaneously visualized within the sampled area and on average 8 ± 2 fluorescent cells are clearly detected in each field (n = 29 fields from 6 different series of cultures, 2D-PDMS and 3D-PDMS, respectively). Fig. 3a (left panels) shows the spatial resolution of measured cells that could be simultaneously traced within the same field of view, with single cell resolution.

At 8 ÷ 12 DIV neurons are usually synaptically connected and display spontaneous activity including bursts emerging by irregular synchronized firing epochs^14^,^15^.

In our recordings, spontaneous Ca^2+^ activity is detected in 41% (36 out of 88 neurons; 2D-PDMS) and, similarly, in 53% (77 out of 144 neurons; 3D-PDMS cultures; Fig. 3b) of the cells visualized in each field. In Fig. 3a (middle panel) examples of fluorescent recordings of active cells are depicted and compared between 2D- and 3D-PDMS cultures (2 representative cells in each field). Episodes of activity are usually represented by spontaneous bursts of activity fully blocked by TTX (1 μM) application (n = 32 neurons, 2D-PDMS and 3D-PDMS; not shown). We measure the occurrence of spontaneous Ca^2+^ episodes in active cells by quantifying the inter-event-interval (IEI) that is significantly (p < 0.001) shorter in 3D-PDMS cultures (33 ± 11 s, n = 77 cells) when compared to 2D-PDMS ones (73 ± 10 s, n = 36 cells; plot in Fig. 3c), thus suggesting a different functional organization due to 3D features of neuronal connectivity.

In a second set of experiments we pharmacologically block GABA_A_ receptors by bicuculline (20 μM; 20 min) application. This antagonist of inhibitory connections is known to alter network activity patterns^33^,^34^, in fact in both groups, in respect to spontaneous activity, the addition of bicuculline significantly decreased the average IEI of episodes detected in 2D-PDMS (p < 0.01) or in 3D-PDMS conditions (p < 0.001). These data are summarized in the plot in Fig. 3c.

In Fig. 3a (right panel), fluorescence tracings show the appearance of Ca^2+^ episodes brought about by bicuculline in active cells that, on average, display IEI of 10 ± 6 s in 3D-PDMS (n = 77 cells; summarized in the plot in Fig. 3c), a value significantly (p < 0.001) lower when compared to that of 2D-PDMS (40 ± 14 s, n = 36 cells). The amount of active cells within each field is not changed by bicuculline application (43% and 55%, respectively 36 out of 84 neurons in 2D-PDMS and 77 out of 140 neurons in 3D-PDMS), and, in a separate sample of 2D and 3D cultures, we could not detect changes in the density of excitatory synapses (see Fig. 4 in Supplementary Information). According to the neuronal nature of such disinhibited episodes, further application of TTX completely removed all Ca^2+^ activity (n = 28 neurons, 2D-PDMS and 3D-PDMS; not shown).

A distinct feature of network dynamics is the emergence of synchronization linking spontaneous or bicuculline-induced calcium bursts detected from different cells located within the same visualized field. We quantify this parameter by measuring the cross-correlation function (see Methods). The measured cross correlation function (CCF) obtained before and after bicuculline applications are not significantly correlated with cell-cell distance (see Fig. 1a in Supplementary Information). The plot in Fig. 3d shows that the mean CCF of spontaneous calcium episodes in 2D-PDMS cultures is of 0.29 ± 0.12 (n = 11 fields), significantly (p < 0.01) smaller than that detected in 3D-PDMS cultures 0.46 ± 0.15 (n = 18 fields), indicative of a lower level of synchronization in 2D-PDMS cultures, regardless the similar amount of active cells. When we force the network to produce the bicuculline-driven activity, in 2D-PDMS cultures the CCF value is not changed (0.31 ± 0.14); on the contrary, in 3D-PDMS we detect a significant (p < 0.001) increase in correlation among the cells analysed (CCF of 0.71 ± 0.22; Fig. 3d). Thus 3D cytoarchitecture affects the self-organization of the neuronal network and its emerging activity.

### Upgrading scaffolds at the nanoscale: the impact of interfacing MWCNTs

In this set of experiments we incorporated nanoscaled architecture in the 3D-PDMS scaffolds by means of MWCNTs, a particularly interesting nanomaterial in engineering electrically propagated tissues^35^. Carbon nanotubes have been shown to impact the assembly of neuronal circuits in culture^15^. The size, high electrical conductivity, and large surface area of carbon nanotubes favour their interactions with distal dendrites, promoting the emergence of improved tissue functions^15^,^36^. We exploit the MWCNTs ability to impact neuronal networks in 3D, by growing neurons in 3D-PDMS scaffolds containing MWCNTs (named 3D-MWCNT; see SEM in Fig. 1c (v) and inset) and the emergent network dynamics is monitored by imaging Ca^2+^ activity compared to that of neurons grown on 2D-MWCNT^13^,^14^,^15^. Fig. 3e shows sample fluorescent tracings from active cells, notably in both 2D- and 3D- MWCNT around virtually all (≥97%) of the visualized cells (109 out of 112 in 2D-MWCNT substrates; 141 out 144 in 3D-MWCNT scaffolds; n = 6 different culture series; histograms in Fig. 3f), are active and generate spontaneous Ca^2+^ episodes. As shown in the histograms of Fig. 3g the mean IEI values were similar between the two groups (15.3 ± 9.5 s, n = 109 cells 2D-MWCNT; 13 ± 5.7 s, n = 141 cells 3D-MWCNT), however both these values are significantly (p < 0.001) different from those measured from 2D-PDMS and 3D-PDMS cultures. Fig. 3h also reports the mean CCF values, similar under the two conditions (2D-MWCNT 0.86 ± 0.08, n = 14 fields; 3D-MWCNT 0.85 ± 0.13; n = 18 fields), but significantly (p < 0.001) higher when compared to 2D-PDMS and 3D-PDMS values. The distribution of CCF when plotted against cell distance is not different from controls (see Fig. 1b in Supplementary Information).

As shown in Fig. 3g, upon long-term perfusion of bicuculline episodes tend to occur at a similar (2D-MWCNT) or slightly faster (3D-MWCNT; not significant) pace, when compared to the respective (2D- and 3D-MWCNT) spontaneous ones. In the presence of bicuculline, Ca^2+^ episodes display IEI of 17.5 ± 8.3 s in 2D-MWCNT cultures (n = 109 cells) and of 8.2 ± 3.4 s in 3D-MWCNT (n = 141 cells), a value significantly (p < 0.01) lower when compared to bicuculline treated 2D-MWCNT. Differently from what observed in the absence of MWCNT, CCF mean values in 2D-MWCNT and 3D-MWCNT were extremely high either in standard solution and when measured in the presence of bicuculline (CCF 0.83 ± 0.09 in 2D-MWCNT and 0.87 ± 0.13 in 3D-MWCNT). Thus interfacing to MWCNTs highly improved cross synchronization of active cells^14^ in both 2D and 3D.

Summarizing our results, the current study shows that there is a significant difference between neurons grown on a flat surface and those grown in 3D structures, as well as between neurons grown in the absence or presence of MWCNTs. In addition, when neurons are interfaced to MWCNTs network activity is similarly boosted both in 2D and 3D, however 3D architecture can still change IEI values in disinhibited activity.

### Modelling the impact of 3D constructs on network activity

We modelled the 2D and 3D network topologies to elucidate the underlying mechanisms determining the different responsiveness observed in the experiments. For the simulations, the networks are built adopting common constructive rules for both 2D and 3D geometries. First rule (Ru1), neurons develop similarly for the 2D/3D topologies, this is translated in setting the same connectivity probability function (i.e. σ^2^ value, see Methods). Second rule (Ru2), the number of synaptic contacts is the same in the 2D/3D topologies. This latter hypothesis is motivated by: i) our intent to investigate the 3D topology impact on network dynamics minimising additional network changes besides those due to the 3D interactions among the constituent elements and ii) the observed similar percentage of active cells in 2D and 3D fields recorded *in vitro* when cells are not exposed to MWCNTs, suggests a comparable number of synaptic contacts^37^.

**Figure 4:**
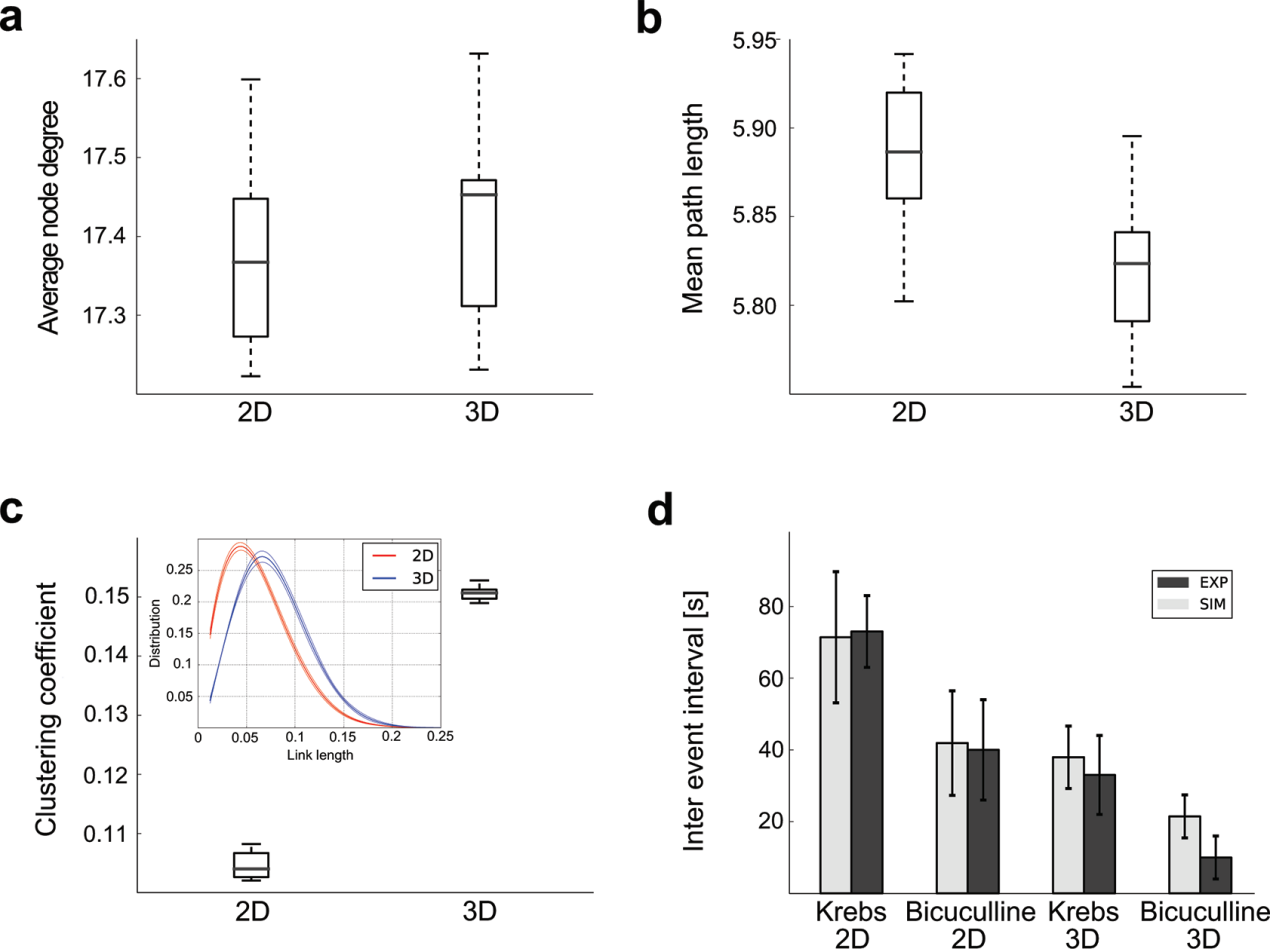
Computational Modelling. Quantification of graph theory and network dynamics parameters of the 2D- and 3D-topologies. The Average node degree (a), Mean path length (b), Clustering coefficient (c) and Inter event intervals (d) are represented under control and bicuculline conditions are represented.

The procedure we use to compare the emerging functions of the 2D/3D network simulations is as follows. First, the 3D networks are built. Second, the 2D networks are obtained according to a two steps procedure: a) the same σ^2^ of the 3D networks is used (c.f. Ru1) b) a set of links of the formed 2D networks is removed to maintain an equal (to the 3D) number of synaptic contacts (c.f. Ru2, see also Fig. 4a). In fact, in a 2D topology the same number of neurons determines a higher cell density on the plane that would cause a higher connectivity level respect to the 3D topology. The removal of the links was constrained to maintain the same distribution of link lengths as well as the same number of connections among the neurons (i.e. synapses, c.f. Ru2).

Multiple runs (n = 10) are performed for each tested 2D/3D topology. The resulting simulated 2D/3D networks display comparable results to the experiments in terms of IEI both in control and when simulating network function upon removal of inhibition (i.e. bicuculline treatment; Fig. 4d).

It should be noted that the lower IEI of the 3D respect to the 2D topology holds across different ratios of excitatory/inhibitory neurons (see Fig.3 in Supplementary Information). We chose the ratio 80/20% because it better agrees with our experimental data (Fig. 4d).

To gain insights into the underlying phenomena responsible of the different 2D and 3D IEI observed in the simulations we performed graph theory measurements. Interestingly, we find that the mean path length (Fig. 4b) stays the same while the clustering coefficient of the 3D networks is higher than in the 2D ones (Fig. 4c). In addition, the link length distribution (i.e. length of the connections, inset Fig. 4c) of the 3D networks displays a shift to longer distances with a longer right-tail compared to the 2D networks. Therefore, the constitutive rules (Ru1, Ru2) determine the increased clustering coefficient and the longer links of the 3D versus the 2D networks that might explain the higher efficiency (i.e. excitability) of the 3D networks.

## Discussion

Conventional 2D plate cultures provide a widely used and powerful tool to study key features of neurons and neuronal synapses but are a limited model when investigating the interplay between anatomical connectivity and dynamics in neural networks at the so called “Biology's new dimension”^38^. On the other hand, cellular neuroscience developments require simple, reproducible and cost efficient platforms to replicate experimental settings enabling 3D cell growth. Technologies aiming at the development of 3D neuronal networks *in vitro* have been going to a burst in the scientific scene^8^, mostly due to neurobiologists' knowledge of the possible disconnection between neuronal network dynamics under 2D and 3D conditions^1^,^32^. In fact, 3D platforms analysed so far support neuronal networks exhibiting properties and behaviours that differ from their equivalent 2D configurations^7^. However, when comparing network behaviour from 2D to 3D platforms, we felt necessary to constrain network changes to the simple 2D or 3D topology of neuronal interactions. Additionally, neurons that grow on supports, such as multi-layered artificial substrates, are only in part exposed to the 3D environment, due to cell adhesion to the platform(s) in itself during *in vitro* growth^3^. The use of PDMS as inert polymer for generating implantable scaffolds is traditionally accepted^16^ nonetheless advantages and limitations of any suitable material need to be evaluated when micro-fabrication technologies are design towards biological applications^39^. Here, we engineered PDMS supports to permit, as shown by our confocal microscopy reconstructions, a genuine 3D growth of percolating neurons and neuroglia, which allows linking the emerging activity of living networks to the mere 2D or 3D interactions among their constitutive elements. The 2D and 3D networks display basically an equal size and are similarly integrated to glial GFAP-positive cells, but differ for their being projected in 2D or in 3D. We believe that our platform provides the unique opportunity of addressing the impact of 3D geometry *per se* in neuronal networks, regardless of the presence of multiple layers of clustered neurons. We observed differences in activity patterns when recorded by calcium imaging in the two culturing conditions. Certainly, in both networks, episodes of calcium activity, whose neuronal and synaptic nature was supported by TTX experiments and fluorescence microscopy inspection of the recorded fields^40^, were reflecting spontaneous synaptic activity, due to synchronous firing, typical of these preparations^14^, without a resolution at the single action potential or individual post synaptic current levels. However, such bursts are an alternative and recognised measure of network state and dynamics^41^. We speculate that 3D networks, besides displaying a similar degree of active cells when compared to 2D ones, display a higher rate of bursting, under both spontaneous and disinhibited conditions, due to an improved efficiency of neurons and neuronal connections in synchronizing their activity.

We favour the hypothesis that the ability to distribute connections in 3D is the main responsible for such behaviours, as outlined by our mathematical modelling.

We cannot rule out that altered cellular excitability in 3D be, at least in part, responsible for our results. However several pieces of evidence suggest this is not the case. In fact, improving cellular excitability by any means would increase the probability of active cells^37^, but such an increase was not observed under our 3D PDMS condition and changes in the density of excitatory synapses appear not to be primarily involved. Interestingly, the impact of 3D topography on network activity was preserved in the presence of bicuculline, excluding a mere change in excitation/inhibition ratio. This was confirmed when simulations were run altering such a parameter. Conversely, when enriching the scaffolds with MWCNTs, known to have a biomimetic effect in instructing the construction of more synapses^15^, virtually all cells recorded in the 2D and 3D fields are active, displaying a high rate of synchronization regardless of the geometry. However, even under these conditions of boosted network activity, 3D configuration is able to affect the occurrence of disinhibited episodes. Our modelling strengthens our hypothesis.

It is intriguing to note that the observed changes in the networks parameters of the model well matches with a recent study on cell culture development^42^. In this study, cell cultures activities were monitored from DIV 7 to DIV 35 and functional connectivity graphs (i.e. a measure of the mutual statistical influence of an electrode on another one) were obtained at each DIV. Along development the functional graphs displayed an increased clustering coefficient, a comparable mean path length and a right shift in the link length distribution as observed in our 3D versus the 2D network models. Based on these observations we can speculate on the fact that the 3D configuration besides representing a more realistic cell arrangement might also ensure an improved maturation of functional organization. Regardless of the proposed maturation in cell arrangements, in both 2D and 3D cultures the removal of GABA_A_ receptor-mediated inhibition significantly improved the occurrence of Ca^2+^ episodes in the active cells, suggesting a similar developmental role of synaptic inhibition in balancing network excitability. Further studies, beyond the scope of this work, will be directed to investigate the impact of 3D construct on synaptic density and plasticity at further stages of neuronal network maturation *in vitro*^43^.

It is tempting to speculate that our 3D system is an efficient tool to mimic complex network behaviour in simplified *in vitro* models^44^.

When interfaced neurons to MWCNTs in 3D constructs we replicated the ability of carbon nanotubes in improving robustness of signal transfer and synchronization^14^,^15^ in cortical cultured circuits. This novel finding sustains the exploitation of scaffolds, engineered at the nanoscale by carbon nanotubes that, by virtue of the acquired physical features may guide different biological responses^35^,^45^,^46^.

Overall, our approach could provide a versatile platform with the potential to contribute with exciting new possibilities both in *in vitro* and *in vivo* applications. For example, our 3D system can be exploited in analysing functional interactions among region-specific brain cells, or can represent a biocompatible cellular scaffold to be engineered for tissue formation, setting the stage for the development of novel hybrid materials.

## Methods

### PDMS scaffolds and MWCNTs

PDMS micro-sponges are three-dimensional, self-standing, porous, structures fabricated from a sugar crystals conglomerate where interstitial cavities were filled by an elastomer. Spontaneous water dissolution of the sugar scaffold results in a cast PDMS framework constituted of connected empty cavities of controlled shape and dimensions (see sketches in Fig.1a).

The pristine PDMS scaffold was produced through a multi-stage process, modified by^16^: 500 mg of food approved sugar was pass through a No. 60 mash sieve, characterized by a nominal sieve opening of about 250 μm (Sigma Aldrich Co.) and mixed with 20 μL of deionized water until a homogeneously wet material is obtained. The mixture is poured into a silicon mold of the desired shape (25 × 7.5 × 7.5 mm), gently pressed and dried at 65°C for 30 minutes. Commercial PDMS silicon elastomer used for scaffold fabrication (Sylgard® 184 – Down Corning Co.) is prepared mixing 10 parts of elastomer and one part of curing agent having a layer of 2 mm in a glass dish. The cube of dried sugar is placed onto the dish and filled up by vacuum induced percolation with PDMS. Subsequently the cube is cured in an oven at 85°C for 1 hour and then left cooling to room temperature. Peripheral PDMS excess is trimmed away, and the cube is dipped in distilled water and here left overnight to dissolve the sugar. The water driven dissolution of sugar grains produces micro-pores inside the PDMS scaffold of irregular shapes mimicking tissue's irregular geometries. Pore connections are the consequence of the aggregation of sugar grains along their contact points via water driven consolidation process.

MWCNT endowed PDMS scaffolds were prepared from a mixture of functionalized MWNT and sugar. MWCNTs were functionalized using 1,3-dipolar cycloaddition with heptanal and sarcosine at 180°C for 24 h in o-dichlorobenzene (ODCB) as solvent.

Functionalized MWCNTs (15 mg) and sugar (500 mg, sifted as above) were mixed in dry condition and shacked overnight. Later, 20 μL of deionized water were added and the blend was mixed until a homogeneously wet compound was obtained. From hereafter fabrication procedure follows the one described above. Before use both scaffolds were sonicated in distilled water for 20 minutes, dehydrated in ethanol solution (99.5%), and dried in an oven at 65°C for 2 hours.

2D MWCNT-coated substrates were prepared similarly to our previous work based on the 1,3-dipolar cycloaddition^14^,^15^ (optimized MWCNT solution concentration 0.1 mg/mL; final MWCNT film density 7 × 10^−5^ mg/mm^2^). Briefly, MWCNTs (Nanostructured & Amorphous Materials, Inc., Stock#: 1237YJS, outer diameter 20 ÷ 30 nm), used as received, were functionalized using 1,3-dipolar cycloaddition as described above. Ethyl acetate solution of functionalized MWCNTs (0.1 mg/mL) was sprayed on glass coverslips placed on a hot plate at 200°C, then the substrates were heated at 350°C under N_2_ atmosphere to induce the complete re-pristinization of MWCNTs.

### Cell culture

Primary cultures of hippocampal neurons were prepared from postnatal (P2–P3) rat pups as previously reported^13^,^14^,^15^. All procedures were approved by the local veterinary authorities and performed in accordance with the Italian law (decree 116/92) and the EU guidelines (86/609/CE, 2007/526/CE and 2010/63/UE). Animals use was approved by the Italian Ministry of Health. All efforts were made to minimize animal suffering and to reduce the number of animals used. Briefly, hippocampi were isolated from the rest of the brain and upon enzymatic treatment neurons were dissociated by pipetting. Cells were plated on five different substrates: poly-L-ornithine-coated, PDMS coated and MWCNT-coated glass coverslips (2D surface control conditions) and on PDMS and PDMS-MWCNT scaffolds (3D growth conditions). Since we did not detect differences in any of our measurements among poly-L-ornithine and PDMS coated cultures, results from all these samples are pooled together and collectively named 2D-PDMS.

Before using for culturing, all 3D scaffolds were sliced at 300–400 μm thickness and mounted on the glass coverslips (12 × 24 mm^2^, 0.13–0.16 mm thick, Kindler, EU) by a thin adhesive layer of PDMS cured at 120°C.

One hour prior to plating, PDMS and PDMS-MWCNT scaffolds were treated with an air plasma-cleaner in order to facilitate cell adhesion and at the end sterilized with an UV lamp.

Cultures were incubated at 37°C, in a humidified atmosphere with 5% CO_2_ in culture medium, consisting of MEM (Gibco), supplemented with 35 mM glucose (CarloErba Reagents), 1 mM Apo-Transferrin, 15 mM HEPES, 48 μM Insulin, 3 μM Biotin, 1 mM Vitamin B12 (Sigma-Aldrich) and 500 nM Gentamicin (Gibco). Culture medium was renewed (60%) after two days from seeding and hereafter changed every 2 days^15^. Plating was carried out at a nominal density of 200.000 ± 16.000 cells/ml (n = 4 different series of cultures). Cultures were then used for experiments after 8 ÷ 12 days *in vitro* (DIV).

### Immunocytochemistry, confocal microscopy, image processing and scanning electron microscopy

Cells cultured in 2D and 3D conditions (8 ÷ 12 DIV) were fixed in PBS containing 4% PFA for 20 min, at room temperature (RT). Cells were permeabilized with 1% Triton X-100 for 30 min, blocked with 5% FBS in PBS (blocking buffer) for 30 min at room temperature and incubated with primary antibodies for 30 min. The primary antibodies used were as follows: rabbit polyclonal anti-β-tubulin III (Sigma T2200, 1:250 dilution) and mouse monoclonal anti-GFAP (Sigma-Aldrich, 1:200 dilution). After the primary incubation and PBS washes, neurons were incubated for 30 min with Alexa 594 goat anti rabbit (Invitrogen, dilution 1:500), Alexa 488 goat anti mouse (Invitrogen, dilution 1:500) and with DAPI (Invitrogen, 1:200 dilution) to stain the nuclei. Samples were mounted in Vectashield (Vector Laboratories) on coverslips of 1 mm thickness. Upon immunofluorescence staining, hippocampal cultures were imaged using a confocal microscope (Leica Microsystems GmbH, Wetzlar, Germany). Both 2D and 3D fixed samples were investigated at 20× magnification and serial confocal planes (z-stack) were acquired every 400 nm across the entire 2D (n = 50 z-stacks maximum) and 3D (n = 200 z-stacks maximum) sections. Reconstructions of the images were performed offline using the image-processing package Fiji^47^. To investigate the z-distribution of nuclei in the two culturing conditions profile reconstruction was performed only for the DAPI channel by the image-processing package Fiji.

Detailed image reconstructions (n = 35 ÷ 50 z-stacks acquired every 600 nm) were performed at higher magnification (63×). MWCNTs were visualised by using the reflection mode property during the confocal acquisition. PDMS and MWCNT scaffold morphologies and cell/MWCNTs interaction were qualitatively assessed through scanning electron microscopy (SEM). Images were acquired collecting secondary electrons on a Gemini SUPRA 40 SEM (Carl Zeiss NTS GmbH, Oberkochen, Germany). Bare scaffolds were mounted on conductive double side carbon tape (Ted Pella, Inc., USA) and imaged at 5 keV. Cellular samples were fixed with the same procedure described for immunocytochemistry, but followed by a dehydration process dipping the sample in water/ethanol solutions at progressively higher alcohol concentrations (50%, 75%, 95% and 100% ethanol for 3 minutes each). Samples were dried at room temperature in a N_2_ chamber overnight. Prior to SEM imaging samples were gold metalized in a metal sputter coater (Polaron SC7620).

### Calcium imaging and data analysis

For Ca^2+^ measurements, hippocampal cells were loaded with cell permeable Ca^2+^ dye Oregon Green 488 BAPTA-1 AM (Molecular Probes); 4 mM stock solution of the Ca^2+^ dye was prepared in DMSO (Sigma-Aldrich) and hippocampal cultures were incubated with a final concentration of 4 μM for 20 min at 37°C in the cell culture incubator. The samples were then placed in a recording chamber mounted on an inverted microscope (Nikon TE-200) where they were continuously superfused by a recording solution of the following composition (mM): 150 NaCl, 4 KCl, 2 CaCl_2_, 1 MgCl_2_, 10 HEPES, 10 glucose (pH adjusted to 7.4 with NaOH; osmolarity 300 mOsm) at 5 mL/min. Videomicroscopy and Ca^2+^-imaging measurements were carried out at RT. The Oregon Green loaded cultures were observed with a 40× objective (0.6 NA, Nikon, Japan). Recordings were performed from visual fields (120 × 160 μm^2^) containing on average 8 ± 2 neurons. Prior to recording Ca^2+^ signals, we selected the cells under examination by drawing regions of interest (ROIs; Fig. 3a and e) around cell bodies and including as little background as possible. Images were continuously acquired by a Till Photonics Till-Imago system, exciting the Ca^2+^-dye with a 488 nm wavelength light generated by a monochromator device equipped with integrated light source (Polychrome IV, Till Photonics). Excitation light was separated from the light emitted from the sample using a 395 nm dichroic mirror and filter. Images of emitted fluorescence >480 nm were acquired continuously for 2700 s as a maximum (200 ms exposure time) by a cooled slow-scan interline transfer camera (IMAGO CCD camera; Till Photonics) and simultaneously displayed on a colour monitor. We verified that the fluorescence signal remained stable during the recording and we did not observe neither photo-bleaching of the calcium probe nor photo-damage of the neurons, as confirmed by robust responses produced by 100 mM KCl (Carlo Erba, Italy) pulse application at the end of the recording sessions (not shown). Camera was operated on 60 × 80 pixels binning mode. The imaging system was controlled by an integrating imaging software package (TILLvisION; Till Photonics) using a personal computer.

In order to induce rhythmic bursts, 20 μM bicuculline methiodide (Sigma-Aldrich) was bath-applied after 15 minutes recording; at the end of each experiment, 1 μM TTX (a voltage-gated, fast Na^+^ channel blocker; Latoxan) was added to the recording solution to confirm the neuronal nature of the recorded signals.

Recorded images were analysed off-line both with the Clampfit software (pClamp suite, 10.2 version; Axon Instruments) and Igor Pro Software (6.32 A version; WaveMetrics, Lake Oswego, Oregon, USA). Intracellular Ca^2+^ transients were expressed as fractional amplitude increase (ΔF/F_0_, where F_0_ is the baseline fluorescence level and ΔF is the rise over baseline); we determined the onset time of neuronal activation by detecting those events in the fluorescence signal that exceed at least five times the standard deviation of the noise. We then computed the difference between consecutive onset times, to obtain the inter-event interval (IEI). Hence, after obtaining the IEI values from each active cell in the field, data were pooled for all fields recorded under the same experimental conditions and averaged for further comparison.

The correlation between the Ca^2+^ events among all active cells recorded from the same field was assessed by cross-correlation analysis (see also Supplementary Information Fig. 2). The value of cross correlation function (CCF) was used to measure the strength of the correlation between cells, i.e. the relative probability that the peaks of calcium transients took place at the same time in all the cells. These values range between 1 (maximal correlation) and −1 (maximal anti-correlation), and were obtained using Igor Pro.

All data are presented as mean ± standard deviation (SD) of the mean (*n* is the number of cells, if not otherwise indicated). Statistical significance for IEI was calculated as function of 2D/3D and presence or not of bicuculline, the differences among the groups were evaluated with Two-way ANOVA followed by the Holm–Sidak test for multiple comparison procedures (Sigmaplot 12.0 – Systat Software). *Student*'*s-t* test (Statistica 6.0 – StatSoft Italy) was used for all other significance comparisons. In both cases a value of p < 0.05 was accepted as indicative of statistically significant difference.

### Computational modelling

#### Cell models

The network consisted of excitatory (80%) and inhibitory (20%) cells with a total of 4096 cells. For the sake of numerical efficiency, each cell was modelled as a single compartment and its dynamics were described by the Adaptive Exponential Integrate and Fire neuron model^48^:
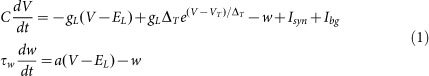


The first equation describes the evolution of the membrane voltage (*V*) of the neuron, where *C* represents the membrane capacity, *g_L_* the leak conductance, *E_L_* the leak reversal potential, *Δ_T_*, *V_T_* regulate the upswing of the voltage, *I_SYN_* is the total synaptic input and *I_bg_* is a background current. The second equation describes the evolution of an adaptive current determining the firing patterns displayed by the neuron, where *τ_W_* is the adaptation time constant and *a* determines the extent of adaption. A spike is generated whenever *V* crosses the threshold *−10 mV*, afterward *V* is reset to *E_L_* and *w* is updated by a constant *b*. A background current (*I_bg_* ~ 100 pA) was delivered to the cells to set the resting potential in the range −60 ÷ −50 mV.

The values of the parameters are reported in Table 1 and in Table 2.

#### Synaptic Transmission

Chemical neurotransmission was implemented by a short term plasticity (STP) mechanism and a synaptic receptor model. The STP model^49^ accounted for synaptic depression following repetitive activation and was characterized by a single exponential time constant (*τ_REC_*) and a release probability (*P_REL_*). The output of the STP model is a variable Y (0 ≤ Y ≤ 1) that corresponds to the available resources (e.g. neurotransmitters). The synaptic conductance (*G*) was modelled as a bi-exponential function:
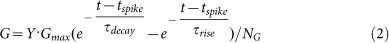
where *G_MAX_* is the maximum conductance value, τ_RISE_ (τ_DECAY_) is the rise (decay) time constant of the synaptic current, *t_SPIKE_* is the instant at which the synapse is activated and the normalization factor *N_G_* guarantees that the maximum of *G* is *G_MAX_*.

The synaptic current was then given by:
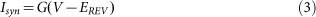
where *E_REV_* corresponds to the reversal potential of the corresponding synaptic current.

Synaptic delays have been included as two components: a fixed amount 0.5 ms, that models neurotransmitter diffusion, the binding and activation of the receptors and a term reflecting the propagation delay (0 ÷ 0.2 ms) of the action potential which is proportional to the distances among the connected cells.

#### Background activity

Sub-threshold synaptic noise was delivered to each cell as independent Poisson processes (mean 1 Hz).

#### Network topology

In the 3D network, neurons were randomly and uniformly distributed in a dimensionless box of size 1 × 1 × 0.25 to reflect the same cellular distribution of the experiments. In the 2D network, neurons were similarly distributed on a planed of size 1 × 1. The connection probability between pairs of neurons was a Gaussian radial basis function (RBF) of zero mean and variance σ^2^ (i.e. highest connection probability for the closest neurons). A directed network was obtained from it by orienting each link with probability 0.5. As a consequence of the connectivity rule, the neurons lying close to the border of the box had a lower number of edges as one might expect it occurs also in the experiments. The value of σ^2^ was chosen such to reproduce network responses comparable to the one observed in cell culture experiments. The adopted σ^2^ (0.0035) resulted in an average in-out degree (i.e. average number of incoming-outgoing connections) ranging from 10 to 20.

#### Network dynamics

The simulated networks well reproduced the main spiking properties of cell culture networks as measured with MEA (i.e. multi electrode array) recording systems. We analyzed network statistics commonly found in the literature^48^. In particular, we considered the mean firing rate (MFR, i.e. number of spikes per second), the mean burst duration (MBD) and the mean intra-burst frequency (MFIB, i.e. firing rate during a burst). The quantification is reported in the Supplementary Fig. 2 where it should be noted that the relatively low firing rate (MFR ∼0.1 Hz) in the model is in line with MEA recordings performed at the same age (c.f. Fig. 2A^50^).

#### Analysis

In the model, the *Inter Event Interval* (IEI) was quantified as the time elapsing between the end of a burst and the initiation of a following one. The MFR, MBD and MFIB network statistics were computed as in previous works^50^,^51^.

#### Graph theory measurements

We used graph theory measurements^44^ to quantify the main properties of the 2D/3D graphs. In particular we considered the average node degree (i.e. average number of incoming/outgoing connections per neuron), the clustering coefficient (i.e. a measure of how well connected neurons to a neuron are connected among themself) and the mean path length (i.e. the average shortest path between any neurons in the graph) of the un-directed graphs.

## Supplementary Material

Supplementary Information Supplementary Information and Results

Supplementary Information Supplementary Movie S1

Supplementary Information Supplementary Movie S2

## Figures and Tables

**Table 1:** Cell parameters

	a (nS)	b (pA)	C (pF)	g_L_ (nS)	E_L_ (mV)	V_T_ (mV)	Δ_T_ (mV)	τ_W_ (ms)
Inhibitory	2 ± 0.005	0	200 ± 0.497	10 ± 0.024	70 ± 0.173	50 ± 0.123	2 ± 0.005	30 ± 0.073
Excitatory		60 ± 0.150	281 ± 0.711	12 ± 0.025				300 ± 0.759

**Table 2:** Synaptic parameters

	Presynaptic		Synaptic Receptor			
	P_REL_	τ_REC_ (ms)	G_MAX_ (nS)	τ_RISE_ (ms)	τ_DECAY_ (ms)	E_REV_ (mV)
AMPA	0.5	256	12	1	3	0
GABA	0.5	512	18	1	8	−70
