# Supplementary material for: From 2D to 3D: novel nanostructured scaffolds to investigate signalling in reconstructed neuronal networks
Source: Sci Rep. 2015 Apr 24;5:9562. doi: 10.1038/srep09562 (PMC5407555; doi:10.1038/srep09562)
Supplement: Supplementary Information — and Results [file srep09562-s1.pdf]

Supplementary Information

From 2D to 3D: novel nanostructured scaffolds to investigate signalling in reconstructed neuronal networks

Susanna Bosi[1§], Rossana Rauti[2§], Jummi Laishram[2], Antonio Turco[1], Davide Lonardoni[3], Thierry Nieus[3], Maurizio Prato[1*], Denis Scaini[2,4*]and Laura Ballerini[2,5*]

[1]Department of Chemical and Pharmaceutical Sciences, University of Trieste, Trieste-Italy; [2]Life Science Department, University of Trieste, Trieste-Italy; [3]Department of Neuroscience and Brain Technologies Italian Institute of Technology (IIT), Genoa-Italy; [4]ELETTRA Synchrotron Light Source, Trieste – Italy; [5]International School for Advanced Studies (SISSA), Trieste-Italy.

[§] These authors equally contributed to the work
* Corresponding authors:
Laura Ballerini, MD International School for Advanced Studies (SISSA) via Bonomea 265 34137 Trieste-Italy email: laura.ballerini@sissa.it
Maurizio Prato prato@units.it ; Denis Scaini dscaini@units.it;

Supplementary information

Supplementary methods

Carbon nanotube purification and functionalization
Commercial MWNTs (Nanoamor Inc., Stock# 1237YJS, 100 mg) are suspended in 100 mL of ODCB and sonicated in a water bath for 20 min. Sarcosine (600 mg, 6.73 mmol) and heptanal (588 mg, 5.14 mmol) are added portion-wise over 10 hours (1 addition every two hours) and the reaction mixture is heated at 180 °C for other 16 hours. MWCNTs are then washed several times by filtration (pore size = 0.45 μm) with ODCB, Methanol and Ethanol and dried under high vacuum. The amount of organic functionalization determined by thermogravimetric analysis is 13 μmol/mg.

Mechanical characterization of PDMS elastomer scaffolds.
For compressive test, cubic samples of PDMS elastomer scaffolds with a dimension of 5 mm in side were cut. Uniaxial microcompression tests were conducted on a Galdabini SUN 500 apparatus. Before compressive testing, an approximately 0.01 N preload was applied to ensure a complete contact between the sample and the two flat surfaces compressing it. A constant speed (15 μm/s) loading cycle was used with a load limit fixed at 50 N. After limit is achieved, we recorded discharging cycle to point out possible sample plastic deformation. Final peak displacement was about 3 mm. Scaffolds were indented using a 20 mm flat punch using a high sensitivity load cell for data collection (CTCA10K5 – AEP Transducers, Italy). Tests were operated at room temperature in the air. Each representative stress-strain curve was obtained by averaging the results from at least 10 tests. Compression and viscoelastic data were analysed with an approximate solution for flat indentation load–relaxation based on elastic–viscoelastic correspondence as described in literature[52].

Electrical characterization of PDMS elastomer scaffolds
Electrical characterization of 2D-MWCNT substrates was done using a Jandel four point probe head (mod. RM–3000) as reported in our previous papers[53].
3D-MWCNT were electrically characterized using an home-made device constituted by two coplanar, gold coated, flat electrodes of about 1×1 cm^2 in dimensions between which a 5 mm side size cube of 3D-MWCNT scaffold is placed. Sample is squeezed between the two electrodes' surfaces with a constant 1 N load in order to assure optimal contact. Resistances were assessed using a low current source-meter (KEITHLEY 2601 System SourceMeter) at an applied bias

voltage of about 10 V. Time dependency of the resistance was determined every 15 seconds on a 10 minutes long period. All electrical measurements were carried out at room temperature, in air. Time dependent resistance plots were obtained by averaging the results from 10 tests. 3D-MWCNT resistivity was calculated from scaffold's geometrical characteristic and the interpolated value of resistance at t=0.

Synaptic density analysis
For comparing glutamate-receptor mediated (i.e. excitatory) synapse densities between the two different growth conditions, 2D-PDMS and 3D-PDMS hippocampal cultures (8 DIV) were immunostained for the vesicular glutamate transporter 1 (VGLUT1) and counterstained for neuronal cytoskeletal component β-tubulin III. The following primary antibodies were used: rabbit polyclonal anti-β-tubulin III (Sigma T2200, 1:250 dilution) and guinea-pig polyclonal anti-vesicular glutamate transporter (Millipore AB5905, dilution 1:2000). After the primary incubation, samples were incubated with the secondary antibodies: Alexa 594 goat anti rabbit (Invitrogen, dilution 1:500) and Alexa 488 goat anti guinea-pig (Invitrogen, dilution 1:500). Confocal acquisition was performed at higher magnification (63x) to better visualize VGLUT1-positive *puncta* and n= 15÷30 Z-stacks were acquired every 400 nm for both conditions (n=26 fields, 2D-PDMS and 3D-PDMS). Offline analysis was performed using Volocity software (Volocity 3D Image Analysis Software, PerkinElmer, Massachusetts, USA). For each set of experiments the cell images were acquired using identical exposure settings. The regions of interest for the quantification were blindly chosen using the tubulin channel. The images were analysed for the number of VGLUT1 *puncta*. For each analysed field, we acquired a Z-stack of images in order to count, with Volocity software, VGLUT1 *puncta* as 3D objects. The resulting numbers were normalized using the corresponding cellular volume calculated on the base of β-tubulin III staining. The plot shown in Supplementary Fig.4 summarizes the data collected for both conditions: 2D-PDMS and 3D-PDMS display a similar total number of VGLIT1 positive *puncta* (1.1 ± 0.5 SD, 2D-PDMS; 0.98 ± 0.5 SD, 3D-PDMS).

<u>Supplementary Results</u>

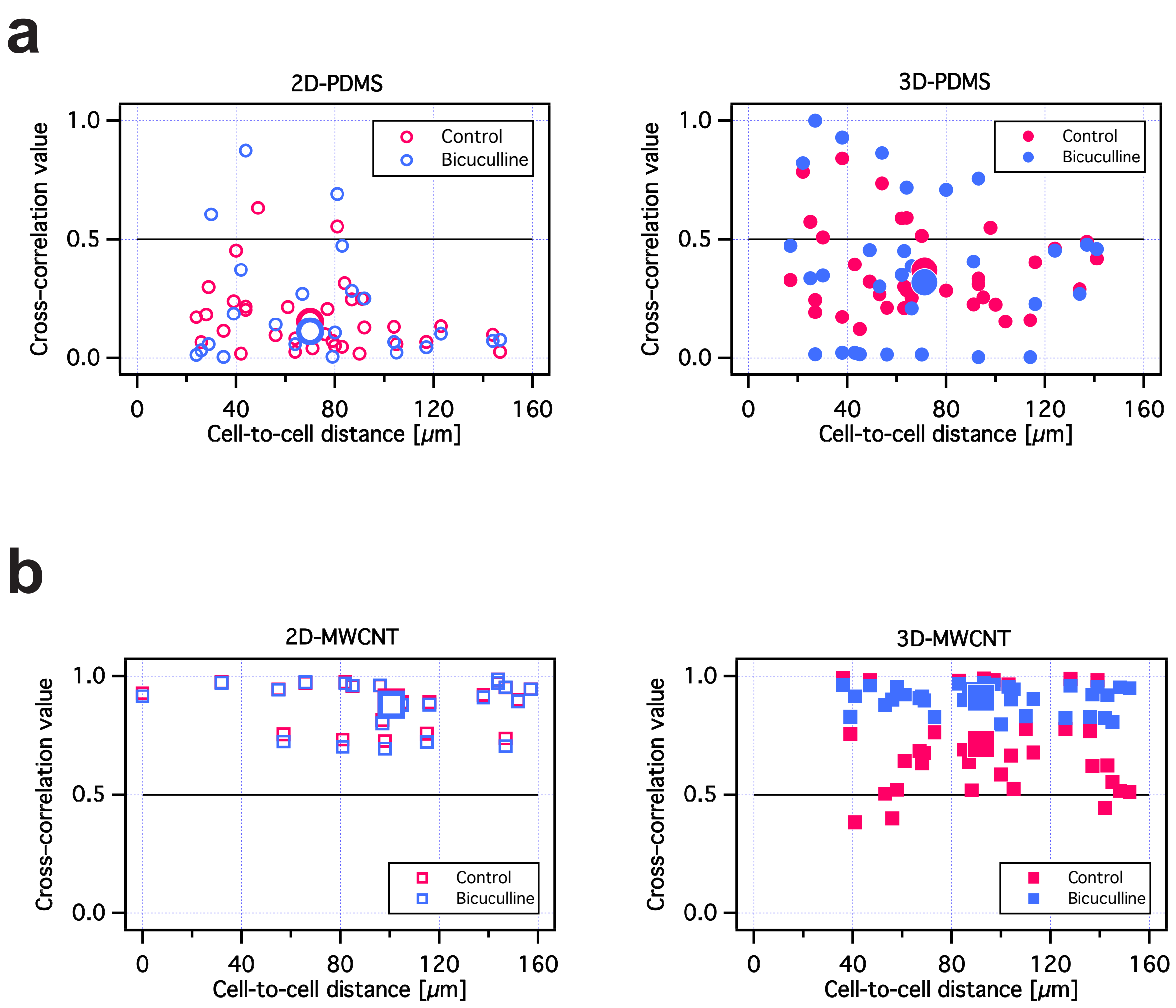

Supplementary information Fig. 1:
Calcium activity in neurons grown under 2D or 3D conditions: cross–correlation analysis.
Pearson's single correlation values for the calcium activity were computed between all the cells present inside a representative 160×120 μm^2 field of view and plotted as function of corresponding cell-to-cell distances. Two cells were considered to correlate or not if their correlation value was larger or smaller of 0.5, respectively. Cross correlation comparison between cellular activity of the bi-dimensional (empty round markers) and three-dimensional (filled round markers) PDMS scaffolds is shown in (a). In (b) correlation data as function of cells' distances are presented for the bi-dimensional (empty square markers) and three-dimensional (filled square markers) MWCNT scaffolds. Correlation values were evaluated before (red markers) and after (blue markers) bicuculline (20 μM) administration. Larger markers represent mean data values. Despite a generally insignificant cell-to-cell distance contribution in both 2D and 3D configurations, carbon nanotubes induce in both systems a remarkable increase in signal correlation.

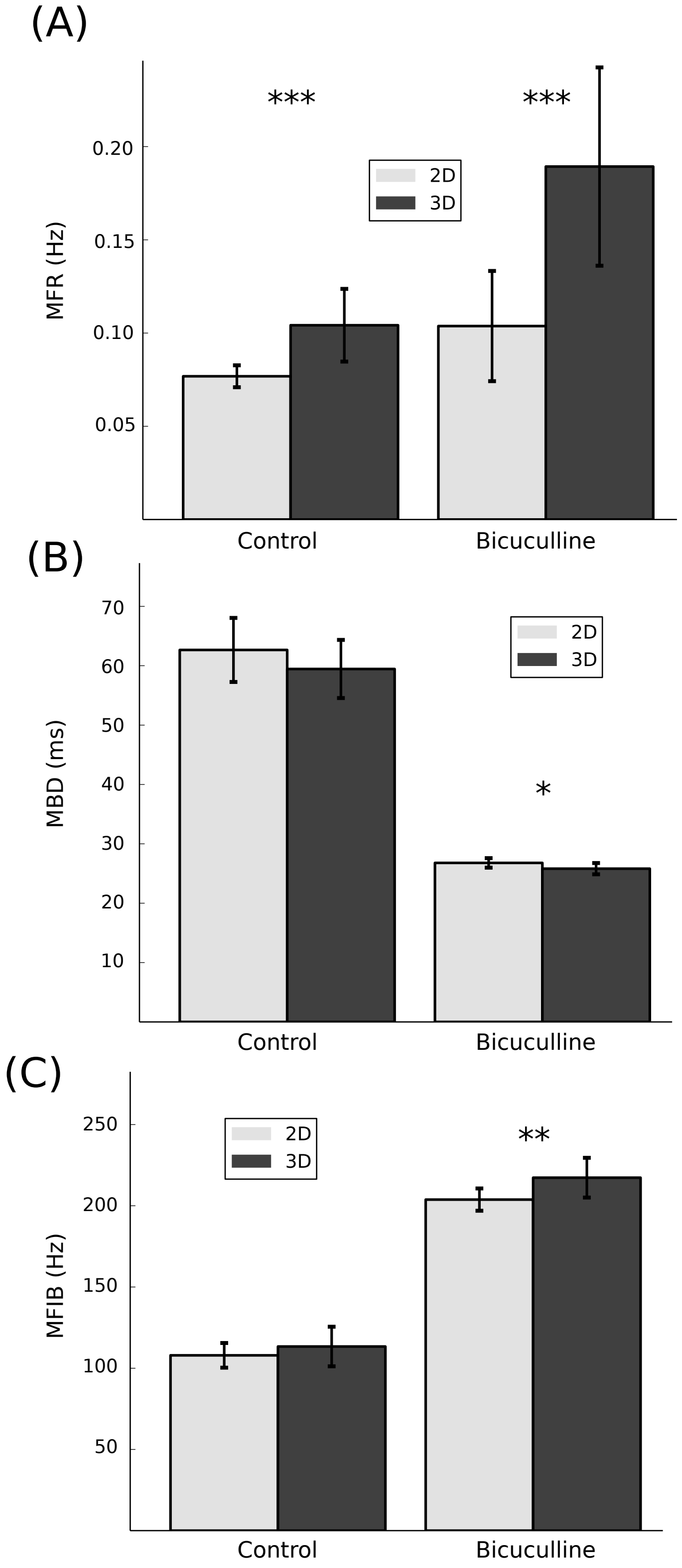

Supplementary information Fig. 2:
Average network dynamics of the 2D versus the 3D model. (A) The MFR increases significantly in the 3D versus the 2D topology, both in the control and bicuculline conditions. (B) The MBD decreases significantly under the simulated bicuculline condition. (C) The MFIB increases significantly under the simulated bicuculline condition. The convention on significance levels is as follows *** $P<0.001$, ** $P<0.01$ and * $P<0.05$ (ANOVA and *Student's-t* test when appropriate).

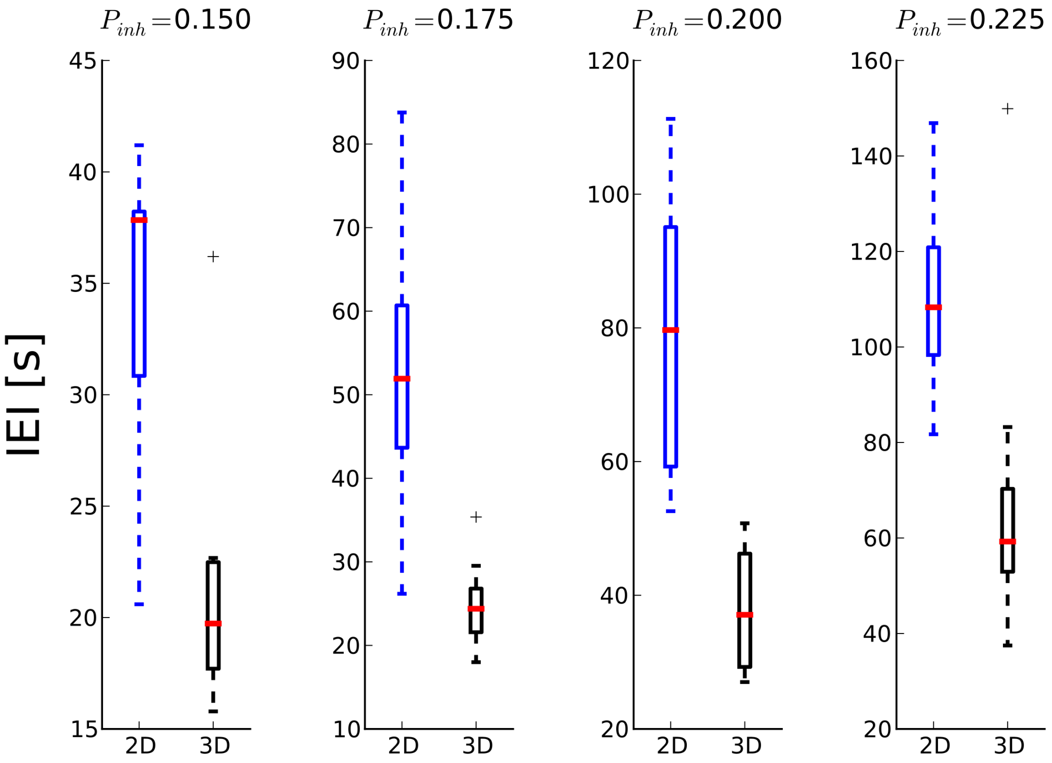

Supplementary information Fig. 3:
Simulated networks at variable ratios of excitatory/inhibitory neurons display a consistent higher IEI in the 2D configuration with respect to the 3D one. The fraction of inhibitory neurons (i.e. P_{inh}) ranges from 0.15 to 0.225 (each boxplot is made of n=8 simulations for each excitatory/inhibitory setting).

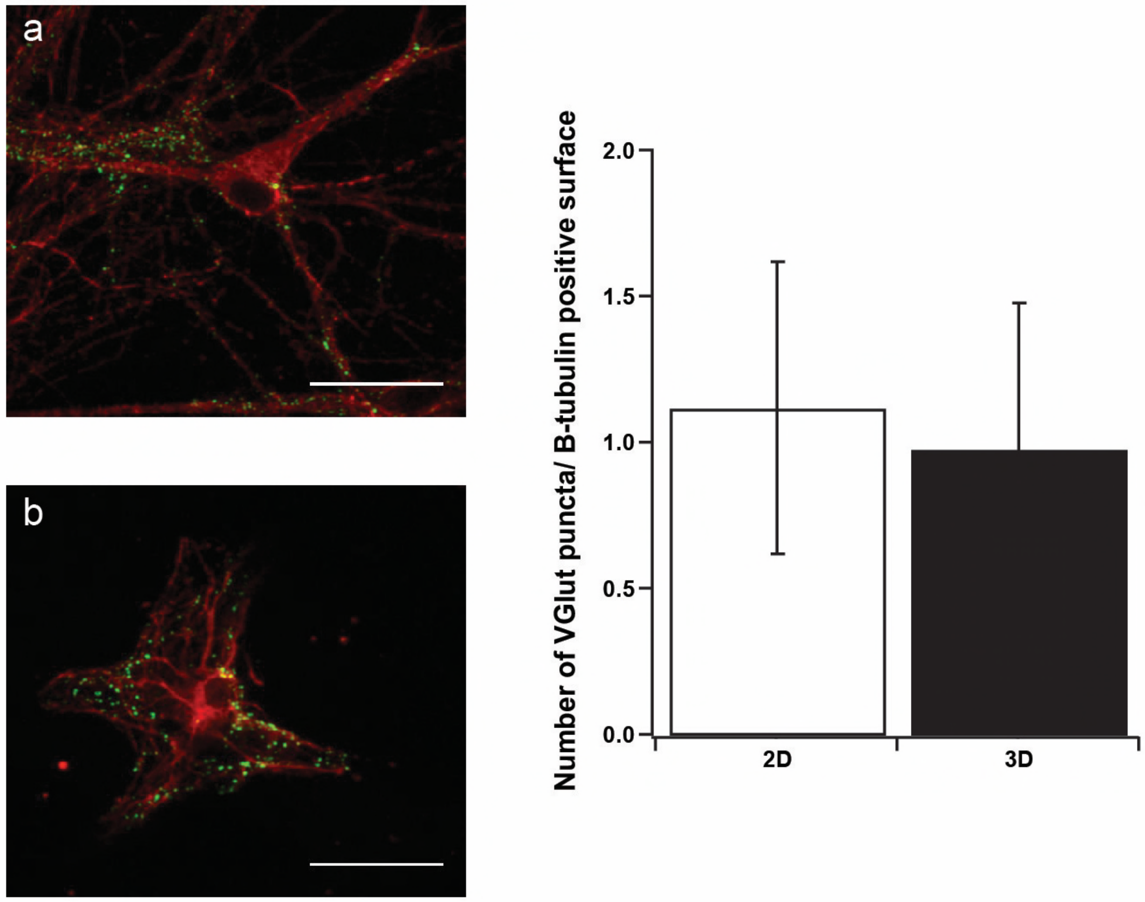

Supplementary information Fig. 4:
Confocal reconstructions (n= 20 Z-stacks) showing hippocampal neurons grown (8 DIV) in 2D-PDMS (a) and 3D-PDMS (b) immunostained for the vesicular glutamate transporter 1 (VGLUT1; green) and counterstained for cytoskeletal components β-tubulin III (red). Scale bar: 50μm. The plot summarizes the result counting VGLUT1 positive *puncta* normalized to β-tubulin positive area (1.1 ± 0.5 SD, 2D-PDMS; 0.98 ± 0.5 SD, 3D-PDMS).

Supplementary information Movie 1:
Confocal 3D reconstruction of hippocampal cultures grown on 3D-PDMS scaffold. β-tubulin-positive neurons (in red) and GFAP-positive neuroglia (green) and DAPI nuclei (blue) are shown. Images were taken for a total thickness of 68.8 μm with a distance between focal planes of 400 nm (n=172 z-stacks). Note the nuclei staining which is visible in different focal planes emphasizing the distribution of cells in a 3D configuration.

Supplementary information Movie 2:
3D hippocampal network investigated at high magnification confocal reconstruction. Images were taken for a total thickness of 22.8 μm with a distance between focal planes of 600 nm (n= 38 z-stacks). β-tubulin-positive neurons (in red) and GFAP-positive neuroglia (green) and DAPI nuclei (blue) are shown. In grey MWCNT visualised by confocal reflecting mode.

Supplementary References

52. Chen, Z. & Diebels, S. Nanoindentation of soft polymers: modeling, experiements and parameter identification. *TECH. MECH.* **34,** 166-189 (2014).

53. Micoli, A. *et al.* Supramolecular assemblies of nucleoside functionalized carbon nanotubes: synthesis, film preparation, and properties. *Chem. Eur. J.* **20,** 5397–5402 (2014).
